# Down-regulating GRP78 reverses pirarubicin resistance of triple negative breast cancer by miR-495-3p mimics and involves the p-AKT/mTOR pathway

**DOI:** 10.1042/BSR20210245

**Published:** 2022-01-06

**Authors:** Mian Liu, Jiu Yang, Wuwu Lv, Shuanglian Wang, Tao Du, Kejing Zhang, Yuhui Wu, Xueping Feng

**Affiliations:** 1 Department of Breast, Xiangya Hospital, Central South University, Changsha, Hunan 410078, P.R. China; 2 Department of General Surgery, Xiangya Hospital, Central South University, Changsha, Hunan 410078, P.R. China; 3 Department of Oncology and Institute of Medical Sciences, Xiangya Hospital, Central South University, Changsha, Hunan 410078, P.R. China

**Keywords:** Chemoresistance, GRP78, miR-495-3p mimics, p-AKT, Pirarubicin chemoresistance, Triple negative breast cancer

## Abstract

Due to the lack of known therapeutic targets for triple-negative breast cancer (TNBC), chemotherapy is the only available pharmacological treatment. Pirarubicin (tetrahydropyranyl Adriamycin, THP) is the most commonly used anthracycline chemotherapy agent. However, TNBC has a high recurrence rate after chemotherapy, and the mechanisms of chemoresistance and recurrence are not entirely understood. To study the chemoresistance mechanisms, we first screened compounds on a pirarubicin-resistant cell line (MDA-MB-231R) derived from MDA-MB-231. The drug resistance index of MDA-MB-231R cells was approximately five times higher than that of MDA-MB-231 cells. MDA-MB-231R cells have higher GRP78 and lower miR-495-3p expression levels than MDA-MB-231 cells. Transfecting MDA-MB-231R cells with a siGRP78 plasmid reduced GRP78 expression, which restored pirarubicin sensitivity. Besides, transfecting MDA-MB-231R cells with miR-495-3p mimics increased miR-495-3p expression, which also reversed pirarubicin chemoresistance. Cell counting kit-8 (CCK-8), EdU, wound healing, and Transwell assays showed that the miR-495-3p mimics also inhibited cell proliferation and migration. Based on our results, miR-495-3p mimics could down-regulate GRP78 expression via the p-AKT/mTOR signaling pathway in TNBC cells. Remarkably, chemo-resistant and chemo-sensitive TNBC tissues had opposite trends in GRP78 and miR-495-3p expressions. The lower the GRP78 and the higher the miR-495-3p expression, the better prognosis in TNBC patients. Therefore, the mechanism of pirarubicin resistance might involve the miR-495-3p/GRP78/Akt axis, which would provide a possible strategy for treating TNBC.

## Introduction

Breast cancer remains the leading cause of cancer-related mortality in women worldwide [1]. The term ‘triple-negative’ breast cancer (TNBC) was coined for cancers lacking detectable estrogen receptor, progesterone receptor, and human epidermal growth factor receptor 2 (HER-2) expression [2]. TNBC accounts for 15– 20% of all breast cancers [3]. Because of this lack of hormone receptors and HER-2 expression, the current TNBC treatment strategies are limited to chemotherapeutic agents, mainly anthracyclines and taxanes. Various targeting agents, such as anti-EGFR drugs, have been studied but have achieved limited success [4]. However, a considerable number of TNBC cases are resistant to chemotherapeutic agents, making them more aggressive and more likely to reoccur than other subtypes and resulting in poorer outcomes [5]. Pirarubicin (tetrahydropyranyl Adriamycin, THP) is a derivative of doxorubicin, which is a commonly used anthracycline chemotherapeutic agent [6]. However, research on pirarubicin resistance in TNBC is lacking.

The glucose-regulated protein 78 (GRP78), also called HSPA5, plays a critical role in stress-induced cellular defense and is a gear in the drug resistance machinery. We previously demonstrated it as one of the radioresistant proteins [7,8]. GRP78 protects cells from apoptosis under stress conditions [9–12]. It is an important biomarker for drug-induced endoplasmic reticulum stress [13]. Besides, GRP78 might play a role in cancer stem cell maintenance and tumor heterogeneity. Furthermore, the possibility of tumor cell resistance to chemotherapy is substantially increased [14–16]. Moreover, strategies targeting GRP78 suppress cell proliferation and enhance gastroenteric cancer and prostate cancer sensitivity to chemotherapeutic agents [17,18]. Limited data are available on the impact of strategies targeting GRP78 on anthracycline resistance and the regulatory molecules involved in TNBC.

MicroRNAs (miRNAs) are noncoding RNAs of approximately 19–25 nucleotides that regulate gene expressions at the post-transcriptional and translational levels by binding to the 3′-UTRs of the target gene and inducing mRNA degradation or protein production repression [19,20]. In tumors, miRNA dysregulation is related to various biological processes, including apoptosis, proliferation, cell cycle progression, epithelial-to-mesenchymal transition, invasion, migration, and metastasis [21]. There is an important correlation between miRNA dysregulation and the response to radiotherapy and chemotherapy [22]. In particular, we previously demonstrated that miR-495-3p played an important role in mediating radiation resistance of nasopharyngeal carcinoma by targeting the 3′-UTR of GRP78, a radiation-sensitive gene [8]. Notably, miR-495-3p is a tumor suppressor down-regulated in prostate cancer, colon cancer, and chemo-resistant gastric carcinoma [23–25]. Moreover, some studies showed that miR-495 was down-regulated in breast cancer tissues and peripheral blood mononucleated cells from patients, making it a molecular signature for breast cancer [26,27]. The role of miR-495-3p in TNBC chemotherapy resistance has not yet been studied and needs further research.

Hence, we evaluated GRP78 and miR-495-3p expression in chemo-sensitive and chemo-resistant TNBC cells and tissues. Moreover, we investigated the roles of GRP78 and miR-495-3p in the response to chemotherapy and the proliferation of chemo-resistant TNBC cells. Finally, we explored the effects of miR-495-3p on GRP78 expression and downstream signaling pathways to provide possible strategies and targets for TNBC treatment.

## Materials and methods

### Cell culture and transfection

We purchased MDA-MB-231 cells from the Cell Bank of Type Culture Collection of the Chinese Academy of Sciences. We developed the resistant MDA-MB-231R cell line in our laboratory (Xiangya Hospital, Central South University) by treating MDA-MB-231 cells with pirarubicin purchased from Zhejiang Haizheng Pharmaceutical Co., Ltd (Zhejiang, China).

We transfected siGRP78 and the miR-495-3p mimics and inhibitors (Guangzhou RiboBio Co., Ltd.) using Lipofectamine 2000 (Invitrogen) according to the manufacturer’s instructions.

### Human tissue specimens

All TNBC patients were admitted to the hospital of Xiangya, Central South University. Inclusion criteria: all patients received at least four neoadjuvant chemotherapy cycles followed by modified radical mastectomy and completed all chemotherapy cycles and radiotherapy assessments after surgery. The response to chemotherapy was evaluated according to the Response Evaluation Criteria in Solid Tumors (RECIST 1.1). Tumors with a total length reduced by 30% or more were considered chemo-sensitive, while tumors with a total length increased by more than 20% were considered chemo-resistant. From January 2011 to December 2015, 12 patients with chemo-resistant TNBC and 12 patients with chemo-sensitive TNBC were identified. The pathology department of Xiangya hospital provided us with paraffin-embedded tissue sections from these patients. In addition, from July 2016 to September 2019, we collected 28 fresh TNBC tissues from patients undergoing TNBC therapy. These patients met the inclusion criteria and tissue collection did not affect their pathological diagnosis. Among the 28 tissue samples, 6 were chemo-resistant and 22 were chemo-sensitive. The fresh tissues were immediately stored at −80 °C. All tissue specimens were certified by the Ethics Committee of Xiangya Hospital, Central South University, China.

### RT-qPCR analysis

Total RNA was extracted from cells and fresh-frozen specimens using Trizol and reverse transcribed using a TransScript® miRNA First-Strand cDNA Synthesis SuperMix kit (TransGen Biotech). RiboBio Co., Ltd. (Guangzhou, China) synthesized the miR-495-3p and U6 primers for miRNA expression analysis. miR-495-3p forward primer: 5′-CGCGTTTGTTTGTACCACGT-3′, reverse primer: 5′-AGTGCAGGGTCCGAGGTATT-3′; U6 forward primer: 5′-GCTTCGGCAGCACATATACTAAAAT-3′, reverse primer: 5′-CGCTTCACGAATTTGCGTGTCAT-3′. Fluorescence-based quantitative real-time polymerase chain reaction (qRT-PCR) was performed using a TransScript® all-in-one miRNA qRT-PCR SuperMix Kit (TransGen Biotech) on a real-time PCR detection system.

### Western blot analysis

Proteins were extracted using a RIPA lysis buffer, and the concentrations were determined using the standard Bovine Serum Albumin (BCA) Protein Assay Kit (Pierce, U.S.A.). Denatured proteins were separated by 8% or 10% SDS-PAGE, then transferred to polyvinylidene fluoride (PVDF) membranes (Millipore, Netherlands), and 5% non-fat milk for 2 h, followed by incubation with primary antibodies overnight at 4 °C and incubation with horseradish peroxidase-labeled secondary antibody for 60 min at room temperature. The protein bands were visualized with super echo-chemiluminescence plus detection reagents and the intensities were quantified using Quantity One software. The dilution rate of the primary antibodies (anti-GRP78 [Abcam, ab21685], anti-AKT [Abcam, ab38449], anti-p-AKT [Abcam, ab8805]) was 1:1000, that of anti-mTOR (Abcam, ab109268) and anti-GAPDH was 1:2000, and that of the secondary antibody was 1:5000.

### Cell Counting Kit-8 (CCK-8) assay

Cell activity and cell proliferation were analyzed using a CCK-8 assay: cells were seeded in 96-well plates at 2–3 ×10^3^ cells/well for the growth test, and 4–5 × 10^3^ cells/well for the chemotherapy agent toxicity assay. They were then incubated with 10 μl of CCK-8 (Dojindo, Japan) for 2 h, and the absorbance at 450 nm was measured to calculate cell growth and vitality rates.

### Colony formation assay

TNBC cells (MDA-MB-231 and MDA-MB-231R) were cultured in 6-well plates at 500 cells/well and then treated with pirarubicin (0.1, 0.3, and 0.9 μM) for 24 h. The plates were incubated for 7–10 days and colonies of 50 cells or more were counted under a microscope. Then, cells were fixed with paraformaldehyde and stained with crystal violet and the numbers or rates of colony formation were calculated.

### Cell Immunofluorescence staining

Cells were placed on a coverslip, grown to 80–90% confluence, then fixed with 95% cold alcohol, and permeabilized with 0.2% TritonX-100. Next, they were blocked with concentrated goat serum for 30 min, incubated with anti-GRP78 antibody overnight at 4 °C and with fluorescent secondary antibody for 30 min. Nuclei were stained with DAPI for 10 min, and pictures were acquired with a confocal microscope with the same parameters.

### EDU assay

At 24 h or 48 h after transfection, MDA-MB-231 and MDA-MB-231R cells were cultured in 96-well plates at about 5 × 10^3^ cells per well. We then performed the EdU assay using an EdU kit (Guangzhou RiboBio Co., Ltd.) according to the manufacturer’s instructions. We captured the pictures with a fluorescence microscope with same parameters.

### Wound healing assay

At 24 or 48 h after transfection, MDA-MB-231 and MDA-MB-231R cells were cultured in 6-well plates at about 5 × 10^5^ cells per well. When cell density reached about 95–100%, scratches were produced using a 100 μl pipette tip. The cells were then incubated in DMEM containing 2% FBS for 24 h. The wounded areas were photographed at different times, and the wound healing rate was calculated as follows: healing rate = (wound width at *t* (h) - wound width at 0 h) / wound width at 0 h.

### Transwell assay

At 24 or 48 h after transfection, MDA-MB-231 and MDA-MB-231R cells were suspended in FBS-free DMEM for 12 h, then approximately 4 × 10^4^ cells in 200 μl of medium were added to each upper chamber (Transwell Boyden Chamber, Costar, USA). After 48 h, the cells that had entered the lower surface of the membrane were fixed with 4% paraformaldehyde for 20 min at room temperature and stained with 0.1% crystal violet in 0.1 mol/l borate, photographed using a photomicroscope (five fields per chamber) (BX51 Olympus, Japan). Finally, the cells were counted by an experimenter blind to the experimental conditions.

### Immunohistochemistry

Immunohistochemistry staining was performed on the TNBC paraffin-embedded tissues as described previously [7,8]. Briefly, the tissues were incubated with a primary anti-GRP78 antibody (1:200, Proteintech, No. 11587-1-AP). The immunohistochemistry staining intensity (indicating GRP78 expression in TNBC tissues) was scored and calculated by two pathologists as previously described. Optical density was measured using Motic Fluo1.0 image analysis software.

### Statistical analysis

Each biological experiment was repeated three times, and the data were reported as the mean ± standard deviation. Statistical comparisons were performed using Student’s *t*-test and one-way or two-way analysis of variance (ANOVA). Graphs were produced using Prism 6 (GraphPad Software). *P* values < 0.05 indicated statistical significance.

### Database search

GRP78 targeting by miR-495-3p was predicted using the Starbase (http://starbase.sysu.edu.cn/index.php), miRDB (http://mirdb.org), and TargetScan (http://www.targetscan.org/mamm_31/) databases. GRP78 expression levels were obtained using GEPIA2 of visual data from the Cancer Genome Atlas (TCGA) (https://gepia.cancer-pku.cn/) and the Oncomine database (https://www.oncomine.org). GRP78 and miR-495 expression levels in breast cancer cell lines were obtained from the CCLE database (https://sites.broadinstitute.org/ccle/). The effect of miR-495-3p expression on survival from breast cancer was obtained by searching Kaplan Meier Plotter (http://kmplot.com/analysis/).

## Results

### Pirarubicin-resistant cells derived from pirarubicin-sensitive MDA-MB-231 cells

**Figure 1:**
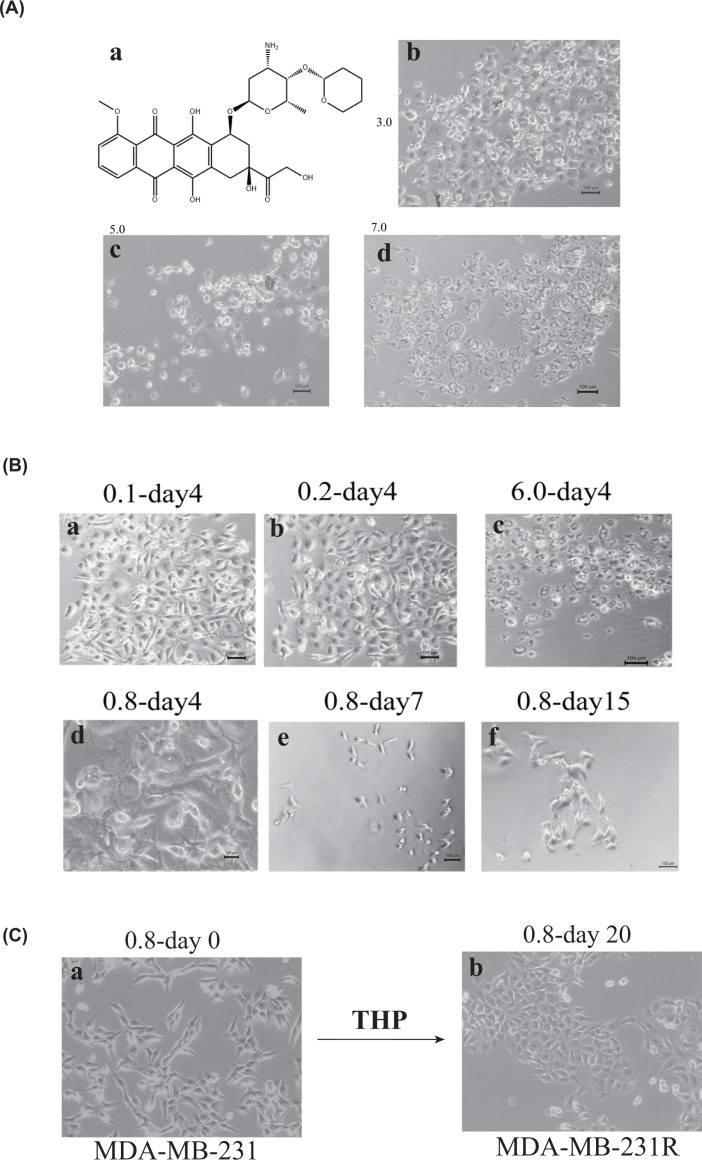
The chemo-resistant TNBC cell line development method (**A**) (a) The molecular formula of the anthracycline pirarubicin. (b) The morphology of MDA-MB-231 cells treated with 3.0 μM THP for 72 h was intact; 6 days later, all cells died. (c) Cells treated with 5.0 μM THP became round and small after 72 h and ultimately died. (d) All cells treated with 7.0 μM THP for 72 h died. (**B**) (a and b) Images of MDA-MB-231 cells treated with 0.1 or 0.2 μM THP for 4 days showing slight cell morphology changes. (c) Images of cells treated with 6.0 μM THP for four days. All cells lost their nuclei and became small; the cell morphology changed, and all cells died. (d–f) Images of cells treated with 0.8 μM THP for 4 (d), 7 (e) and 15 (f) days. After treatment with 0.8 μM THP, cells initially became bigger, then smaller, and finally formed greater numbers of larger cells. (**C**) (a) The parental MDA-MB-231 cells were cultured for 24 h; (b) MDA-MB-231R cells were derived from MDA-MB-231 cells after 20 days of treatment with 0.8 μM THP.

We treated MDA-MB-231 cells with 24 different concentration gradients (0.01–14 μM) of pirarubicin; higher THP concentrations were more toxic (Figure 1). After 48 h, different concentrations of THP exerted different effects on cell morphology, and we observed dose-dependent toxicity. Low concentrations inhibited cell proliferation to a limited extent and caused minor damage to the cell membrane structure. Meanwhile, a high concentration (7 μM) substantially damaged the cell structure, as the cell morphology was no longer visible (Figure 1A), and killed almost all cells (Figure 1A). After 4 days of treatment, all cells treated with 1 μM THP died, but a few of those treated with 0.8 µM survived up to 7 days (Figure 1B). These surviving cells were called subclones, and we defined this concentration (0.8 μM) as the sublethal dose of THP to MDA-MB-231 cells. The surviving cells were potentially resistant to THP., The MDA-MB-231 cell subclones treated with the sublethal concentration proliferated. After treatment with 0.8 μM THP for 20 days, we selected the largest subclone and named it MDA-MB-231R (MDA-MB-231 cells resistant to THP) (Figure 1C).

### MDA-MB-231R cells were more resistant to pirarubicin

**Figure 2:**
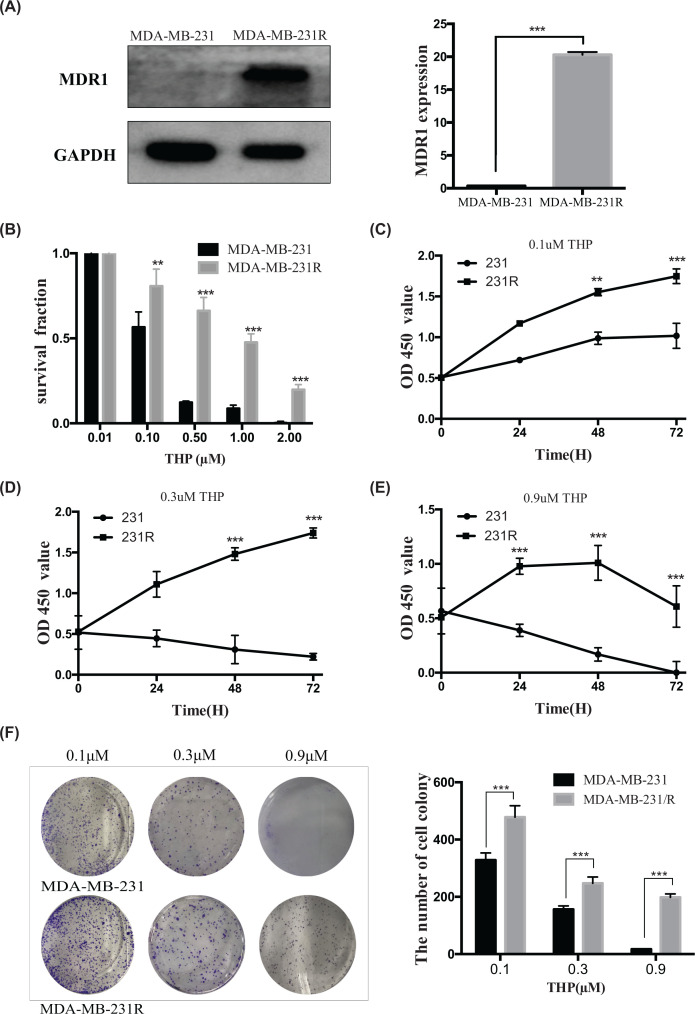
MDA-MB-231R cells were more resistant to chemotherapy than MDA-MB-231 cells (**A**) Western blot showing that MDR1 expression was high in MDA-MB-231R cells and almost non-existent in MDA-MB-231 cells. Left panel: image; right panel: quantification. (**B**) After treatment with 0.01-2.0 μM THP, a higher survival percentage was observed for the MDA-MB-231R subclone than for the parental MDA-MB-231 cells a. (**C**) CCK-8 assay results. MDA-MB-231R grew more than MDA-MB-231 after treatment with 0.1 μM THP. (**D**) Treated with 0.3 μM THP, MDA-MB-231R cells maintained the ability to grow, but MDA-MB-231 cells gradually died. (**E**) MDA-MB-231R cell growth decreased after 48 h of treatment with 0.9 μM THP, and MDA-MB-231 cells rapidly died. (**F**) After treatment with 0.1, 0.3, and 0.9 μM THP, MDA-MB-231R cells formed more clones than MDA-MB-231 cells did. **P<0.01; ***P<0.001.

MDA-MB-231R cells were more resistant to THP than the parental MDA-MB-231 cells (Figure 2). Pirarubicin had a higher IC_50_ in MDA-MB-231R cells (1.296 μM) than in MDA-MB-231 cells (0.274 μM), and the THP resistance index of MDA-MB-231R cells was approximately five times higher than that of MDA-MB-231 cells. In addition, the viability of MDA-MB-231R cells was higher than that of MDA-MB-231 cells after treatment with 0.01–2.0 μM THP (Figure 2B).

Next, we compared the proliferation of MDA-MB-231R and MDA-MB-231 cells treated with 0.1, 0.3, and 0.9 μM THP using CCK-8 and colony formation assays. Treated with 0.1 µM THP, MDA-MB-231R cells proliferated faster than MDA-MB-231 cells (Figure 2C). Treated with 0.3 μM THP, MDA-MB-231R cells still proliferated, while MDA-MB-231 cells gradually died (Figure 2D). Finally, treated with 0.9 μM THP, MDA-MB-231R cells died after 48 h, and MDA-MB-231 cells died faster (Figure 2E). Similarly, MDA-MB-231R cells formed significantly more colonies than the parent MDA-MB-231 cells when treated with 0.1, 0.3, and 0.9 μM THP (Figure 2F).

### MDA-MB-231R cells express high GRP78 levels

**Figure 3:**
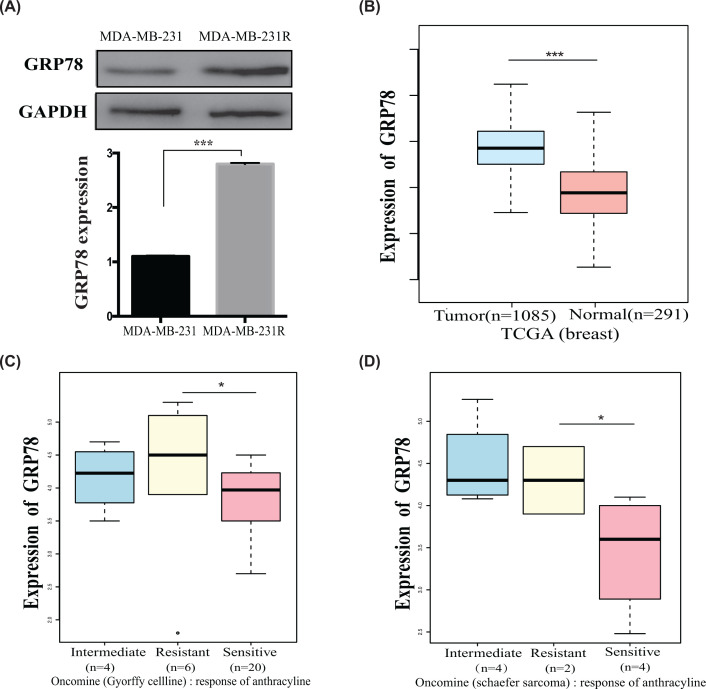
GRP78 expression is high in MDA-MB-231R cells and anthracycline-resistant tumors (**A**) Western blot showing that MDA-MB-231R cells expressed higher GRP78 levels than the parental MDA-MB-231 cells. (**B**) According to the TCGA database, breast cancer tissues express higher GRP78 levels than normal human breast tissue. (**C** and** D**) According to the TCGA (https://gepia.cancer-pku.cn/) and Oncomine (https://www.oncomine.org) databases, GRP78 expression levels were high in anthracycline-resistant cells, intermediate in cells with intermediate anthracycline sensitivity, and low in anthracycline-sensitive cells (**C.** Gyorffy Cell line, **D.** Schaefer sarcoma). *P<0.05; ***P<0.001.

GRP78 is an important biomarker for drug-induced endoplasmic reticulum stress [7], and we previously demonstrated that it was radioresistant [7,8]. Therefore, we investigated its role in the chemotherapy response. MDA-MB-231R-resistant cells expressed higher GRP78 levels than parental MDA-MB-231 cells, as determined by Western blot analysis (Figure 3A). According to TCGA, breast cancer tissues express higher GRP78 levels than normal breast tissues (Figure 3B). In addition, by searching the Oncomine database, we found the highest GRP78 expression levels in chemo-resistant cancer cells, moderate GRP78 expression levels in intermediate chemo-sensitive cells, and the lowest GRP78 expression levels in chemo-sensitive cells such as the Gyorffy cell line and Schaefer Sarcoma research cell line [28,29] (Figure 3C,D). These results indicate a positive correlation between GRP78 overexpression and chemotherapy resistance.

### Down-regulating GRP78 in MDA-MB-231R cells reverses pirarubicin resistance

**Figure 4:**
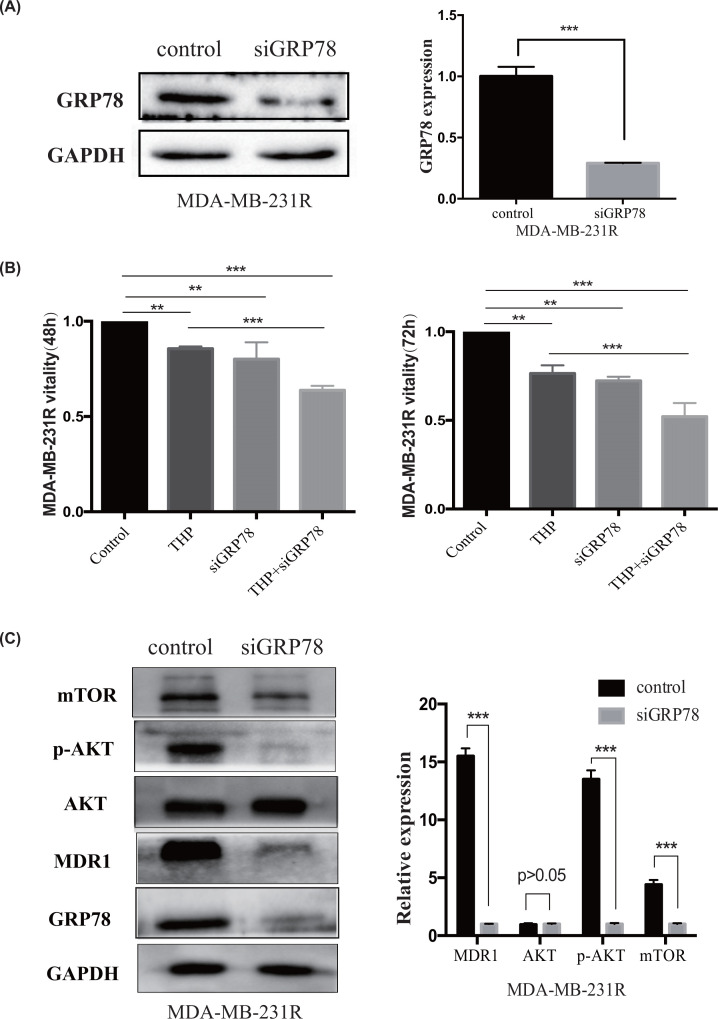
Decreased GRP78 expression reverses pirarubicin resistance in MDA-MB-231R cells (**A**) GRP78 was down-regulated by transfecting MDA-MB-231R cells with the siGRP78 plasmid. (**B**) After 48 and 72 h of treatment with THP and transfection with siGRP78, the viability of MDA-MB-231R cells was lower than that of MDA-MB-231R cells treated with a single reagent. (**C**) Transfecting MDA-MB-231R cells with siGRP78 down-regulated MDR1, p-AKT, and mTOR levels but not the AKT levels. Left panel: Western blot; right panel: quantification. **P<0.01; ***P<0.001.

GRP78 is associated with apoptosis, and down-regulating GRP78 promotes breast tumor cell apoptosis [30]. Hence, we investigated the effect of GRP78 silencing on the efficiency of pirarubicin chemotherapy (Figure 4). We down-regulated GRP78 by transfecting MDA-MB-231R cells with siGRP78 (Figure 4A). Decreasing GRP78 expression in MDA-MB-231R cells enhanced THP efficacy (Figure 4B), as evidenced by the decrease in cell viability and increase in apoptosis. Transfecting the siGRP78 plasmid synergistically increased the response of resistant TNBC cells to chemotherapy; in other words, we reversed the chemoresistance of TNBC cells by down-regulating GRP78 expression.

Furthermore, we analyzed the relationship between GRP78 expression and multi-drug resistance-1 (MDR1) expression in response to THP exposure. The chemoresistance protein MDR1 was only expressed in MDA-MB-231R cells and not in MDA-MB-231 cells (Figure 2A). Interestingly, down-regulating GRP78 significantly reduced MDR1 expression by down-regulating the Akt/mTOR pathway in MDA-MB-231R cells (Figure 4C).

### MiR-495-3p mimics down-regulate GRP78 via the p-Akt/mTOR pathway

As described above, GRP78 plays a role in the chemoresistance of MDA-MB-231R cells via the p-AKT/mTOR pathway. Our following experiments confirmed that the miR-495-3p mimics down-regulated GRP78 through the p-AKT/mTOR pathway.

**Figure 5:**
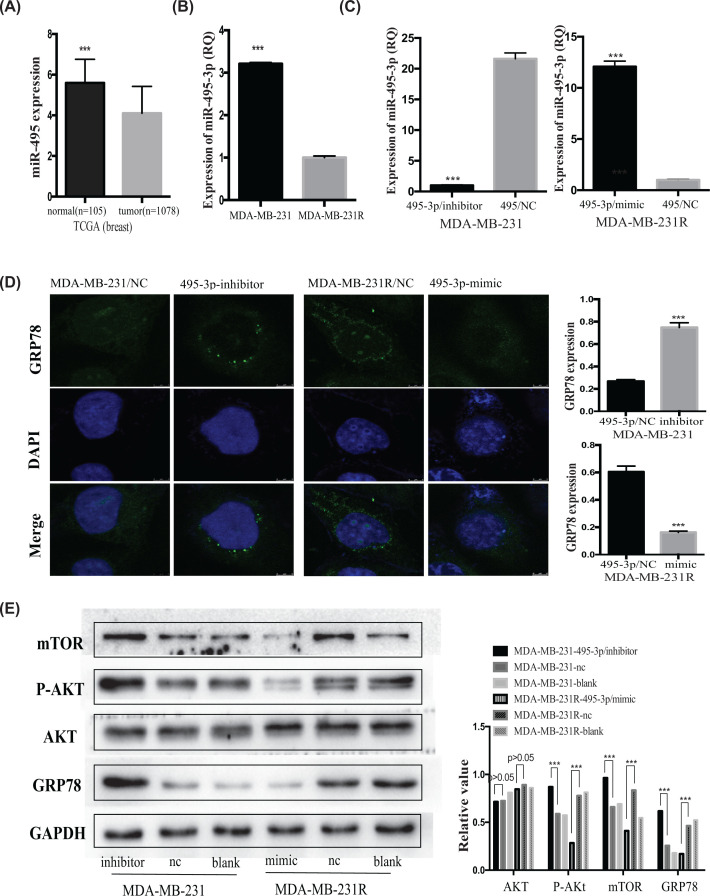
MiR-495-3p down-regulates GRP78 expression via the p-AKT/mTOR signaling pathway (**A**) According to the TCGA database, MiR-495 expression was higher in normal breast tissues than in cancer tissues. (**B**) q-PCR results showed that MDA-MB-231R cells expressed lower miR-495-3p levels than MDA-MB-231 cells. (**C**) Transfecting miR-495-3p inhibitors in MDA-MB-231 cells decreased miR-495-3p expression, while transfecting miR-495-3p mimics increased it. (**D**) Immunofluorescence staining results showing that down-regulating miR-495-3p increased GRP78 (green staining) expression in MDA-MB-231 cells, and overexpressing miR-495-3p down-regulated GRP78 expression in MDA-MB-231R cells. Blue: nuclei (DAPI staining). (**E**) Western blot showing that transfecting miR-495-3p inhibitors in MDA-MB-231 cells increased the GRP78, p-AKT, and mTOR levels, and transfecting miR-495-3p mimics in MDA-MB-231R cells decreased them. Left panel: Western blot; right panel: quantification. ***P<0.001.

First, searching TCGA, we found a negative correlation between miR-495 and GRP78 expression: breast cancer tissues had higher GRP78 and lower miR-495 expression levels than normal tissues (Figures 3B and 5A). Then, by searching the CCLE database, we incorporated data for the negative correlation observed between GRP78 and miR-495-3p in breast cancer cell lines, especially in metastatic cell lines (Supplementary Figure S1). Furthermore, qRT-PCR showed that MDA-MB-231R cells express less miR-495-3p, a regulator of GRP78 [8,31] than MDA-MB-231 cells (Figure 5B). According to the results from the miRDB and TargetScan databases, miR-495-3p should target the GRP78 3′-UTR (Supplementary Figure S2). Besides, we previously confirmed that miR-495-3p targets the GRP78 3′-UTR [8].

Then, we showed that silencing miR-495-3p expression by transfecting inhibitors increased GRP78 expression and that over-expressing miR-495-3p by transfecting mimics down-regulated GRP78 expression in MDA-MB-231R cells (Figure 5C–E). These results suggested that miR-495-3p down-regulated GRP78 expression and the changes in GRP78 expression followed those of the miR-495-3p expression (Figure 5C–E). Besides, down-regulating miR-495-3p and over-expressing GRP78 promoted AKT phosphorylation (p-AKT) and mTOR expression in MDA-MB-231 cells (Figure 5E). However, up-regulating miR-495-3p and down-regulating GRP78 both inhibited the p-AKT/mTOR signaling pathway in MDA-MB-231R cells (Figure 5E). According to a previous study, membrane-associated GRP78 expression correlated with p-AKT levels in pancreatic ductal adenocarcinoma [32].

In the present study, miR-495-3p decreased GRP78 expression via the p-AKT/mTOR pathway. Down-regulating GRP78 using miR-495-3p mimics reversed the chemoresistance of TNBC cells.

### MiR-495-3p mimics inhibited the proliferation, migration, and pirarubicin resistance of MDA-MB-231R cells

**Figure 6:**
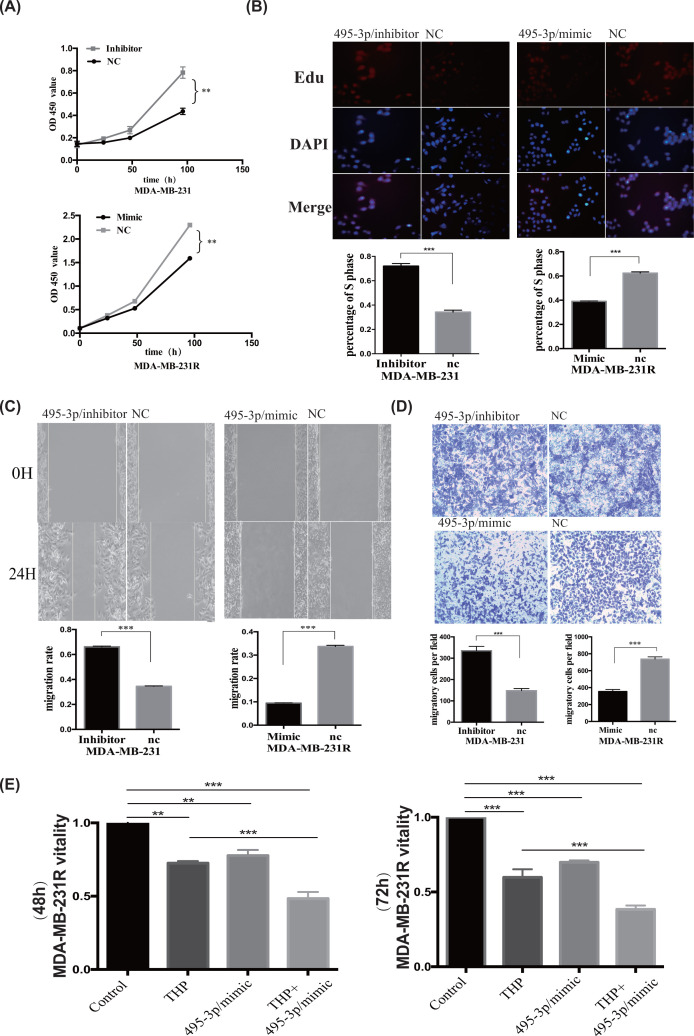
The miR-495-3p mimics decrease pirarubicin resistance and inhibit proliferation and migration in MDA-MB-231R cells (**A**) CCK-8 assay results showing that the miR-495-3p mimics inhibited the proliferation of MDA-MB-231R cells and the miR-495-3p inhibitors promoted the growth of MDA-MB-231 cells. (**B**) EdU experiment results showing that overexpressing miR-495-3p induced MDA-MB-231R apoptosis, and silencing miR-495-3p reduced the death rate of MDA-MB-231 cells. (**C**) The miR-495-3p mimics suppressed the migration of MDA-MB-231R cells, and miR-495-3p inhibitors promoted it. (**D**) Transwell assay results showing that up-regulating miR-495-3p inhibited the invasion of MDA-MB-231R cells, and down-regulating miR-495-3p enhanced it. (**E**) The miR-495-3p mimics cooperated with THP to enhance the toxicity of THP chemotherapy in MDA-MB-231R cells after 48 or 72 h. **P<0.01; ***P<0.001.

Next, we explored the role of miR-495-3p in chemotherapy response, proliferation, and migration. CCK-8 assays revealed that transfecting miR-495-3p inhibitors significantly increased MDA-MB-231 cell growth, while transfecting miR-495-3p mimics inhibited MDA-MB-231R cell growth (Figure 6A). EdU assays revealed similar effects of miR-495-3p on cell proliferation (Figure 6B). Wound healing and Transwell assays showed that cell migration and invasion were enhanced by down-regulating miR-495-3p in MDA-MB-231 cells but suppressed by over-expressing miR-495-3p in MDA-MB-231R cells (Figure 6C,D). Moreover, miR-495-3p mimics increased THP efficiency on MDA-MB-231R cells (Figure 6E). Similar to siGRP78, miR-495-3p mimics cooperated with chemotherapy agents and enhanced tumor suppression. Overall, miR-495-3p inhibited TNBC cell growth and migration and restored chemotherapy sensitivity.

### GRP78 expression negatively correlates with miR-495-3p expression in chemo-resistant TNBC tissues

**Figure 7:**
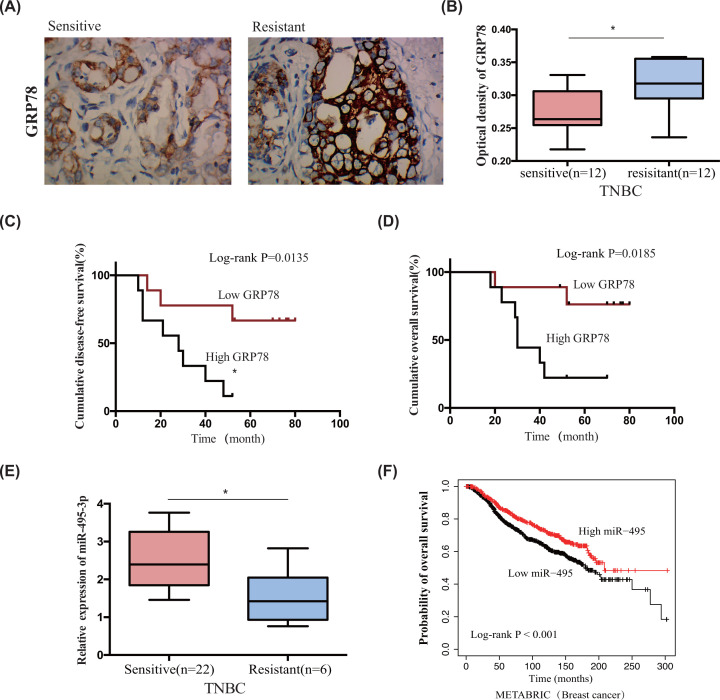
Increased GRP78 expression and decreased miR-495-3p expression in chemo-resistant TNBC correlate with a poor prognosis (**A**) Immunohistochemical staining for GRP78 was strong in chemo-resistant tissues and weak in chemo-sensitive TNBC tissues. (**B**) The optical density of GRP78 was higher in chemo-resistant TNBC tissues than in chemo-sensitive ones (*P*<0.05). (**C**) A high GRP78 expression was associated with shorter disease-free survival (DFS), and a low GRP78 expression was associated with longer disease-free survival (log-rank *P*=0.0135). (**D**) Low GRP78 expression was associated with prolonged overall survival (OS) (log-rank *P*=0.0185). (**E**) RT-qPCR showed that miR-495-3p expression was lower in chemo-resistant tissues than in chemo-sensitive tissues (*P*<0.05). (**F**) High miR-495-3p expression correlated with longer overall survival for patients with breast cancer in the METABRIC database (log-rank *P*<0.001).

We measured the expression patterns of GRP78 and miR-495-3p in chemo-sensitive and chemo-resistant TNBC tissues using immunohistochemistry and qRT-PCR to examine their clinical relevance. Table 1 shows the clinicopathological features of the patients. Chemo-sensitive TNBC tissues had lower GRP78 expression levels and significantly higher miR-495-3p expression levels than chemo-resistant tissues (Figure 7A,B,E). Notably, patients in the high GRP78 expression group exhibited shorter disease-free survival and overall survival than patients in the low GRP78 expression group (Figure 7C,D). However, breast cancer patients in the METABRIC database with high miR-495-3p expression levels had a better prognosis than patients with low expression levels (Figure 7F).

**Table 1:** Clinicopathological features of TNBC

Item	GRP78		*P* value	miR-495-3P		*P* value
	High (*n*=14)	Low (*n*=10)		High (*n*=18)	Low (*n*=10)	
**Chemo-response**			0.0361			0.0126
**Sensitive**	4	8		17	5	
**Resistant**	10	2		1	5	
**T stage**			>0.05			>0.05
**1-2**	8	8		11	4	
**3-4**	6	2		7	6	
**N stage**			>0.05			>0.05
**0**	3	3		6	2	
**1-2**	10	7		12	7	
**3**	1	0		0	1	
**Clinical stage**			>0.05			>0.05
**I-II**	10	8		14	6	
**III**	4	2		4	4	
**Chemo-regiment** ^*^			>0.05			>0.05
**AC-P/T**	11	9		13	6	
**TAC/PAC**	3	1		5	4	
**Ki67**			>0.05			>0.05
**≥15**	10	9		14	7	
**<15**	4	1		4	3	
**p53**			>0.05			>0.05
**Positive**	7	4		8	4	
**Negative**	7	6		10	6	

* **chemo regimen**, AC-P/T indicates 4 cycles of anthracycline and cyclophosphamide chemotherapy followed by 4 cycles of paclitaxel or docetaxel. TAC/PAC indicates 6 cycles of anthracycline and cyclophosphamide combined with paclitaxel or docetaxel chemotherapy.

## Discussion

TNBC cells do not respond to hormonal therapy and treatments targeting HER-2 receptors [1,2]. Moreover, the lack of target genes in TNBC limits the targeted therapy options. Thus, it is crucial to find novel genes enabling to restore chemotherapy sensitivity in TNBC.

Pirarubicin is commonly used to treat TNBC. However, Cell Bank contained no pirarubicin-resistant TNBC cell line. Therefore, to find new genes of interest, we first established the chemo-resistant MDA-MB-231 cell line according to our previous study by treating MDA-MB-231 cells with different THP concentrations [7]. We confirmed the pirarubicin resistance of the surviving subclonal cells and named them MDA-MB-231R cells. Their THP resistance index was approximately five times higher than that of MDA-MB-231 cells.

In further experiments, we found that chemo-resistant MDA-MB-231R cells expressed significantly higher GRP78 levels than the parent MDA-MB-231 cells. Besides, we previously identified GRP78 as a radioresistant protein [7,8]. GRP78 is an important drug-induced endoplasmic reticulum stress radiomarker [33]; it promotes proliferation, inhibits apoptosis, migration, and invasion, alters metabolism, and promotes other malignant behaviors, further increasing chemotherapy resistance in various carcinomas [34,35].

In the present study, chemo-resistant MDA-MB-231R cells and chemo-resistant TNBC tissues significantly over-expressed GRP78 compared with chemo-sensitive MDA-MB-231 cells and chemo-sensitive TNBC tissues. Notably, the GRP78-mediated epithelial–mesenchymal transition (EMT) plays an important role in inducing and maintaining the cancer stem cell phenotype and the protective mechanism of cells, enabling tumors to escape attacks and survive chemotherapy or radiotherapy [16,36–39].

Knocking down GRP78 increased chemo-sensitivity in cancer stem cells and MCF-7 cells, and the higher the transfection efficiency, the better the chemotherapy response [40]. Silencing GRP78 by diminishing the antioxidant response and decreasing the efflux activity of ATP-binding cassette transporters may deregulate the chemoresistance of pancreatic cancer [17]. In the present study, the elevated GRP78 increased the chemoresistance of MDA-MB-231R cells, consistent with previous studies. Furthermore, up-regulating GRP78 increased the chemotherapy resistance of TNBC, while down-regulating GRP78 using siGRP78 restored the chemo-sensitivity of MDA-MB-231R cells. Therefore, these observations revealed that GRP78 is a chemoresistance gene and might play a vital role in the progression of drug-resistant TNBC.

In gastric cancer, miR-495-3p inhibits multidrug resistance by modulating autophagy through the GRP78/mTOR axis [25]. Furthermore, our data showed that miR-495-3p-mediated GRP78 down-regulation was related to p-AKT/mTOR signaling. These results suggest that miR-495-3p and GRP78 are important chemotherapy sensitivity mediators in MDA-MB-231R cells. Notably, miR-495 exerts antitumor effects and inhibits the progression of lung cancer, prostate carcinoma, colon cancer, gastric cancer, oral cancer, and leukemia [23–25,41–43].

Moreover, there is a positive relationship between GRP78 expression and the expression of the antiapoptotic protein Bcl-2, and down-regulating GRP78 promotes apoptosis in breast tumor cells [30]. In non-small cell lung cancer and gastric cancer, miR-495 targets the 3′-UTR of GRP78, which plays a causative role in tumorigenesis and regulates chemoresistance [25,44,45]. Our previous study showed that miR-495 enhanced the efficacy of radiotherapy in nasopharyngeal carcinoma cells by targeting the 3′-UTR of GRP78 [8]. In our current study, MDA-MB-231R cells and chemo-resistant tissues expressed low miR-495-3p levels.

In TNBC, miR-495-3p and GRP78 expressions follow opposite trends. Besides, the change in GRP78 levels followed the change in miR-495-3p levels. In addition, a high miR-495 expression and a low GRP78 expression were associated with a better prognosis. Fei and colleagues have shown that up-regulating microRNA-495 inhibits the PI3K/AKT signaling pathway [46]. Moreover, miR-495 could reverse cisplatin resistance in non-small cell lung cancer by regulating the expression of the drug resistance genes ABCG2 and ERCC1 and directly targeting UBE2C 3′-UTR, affecting cells growth, invasion, and metastasis [47]. These results are consistent with our conclusion that transfecting the miR-495-3p mimic into MDA-MB-231R cells enhanced the sensitivity of TNBC to pirarubicin by down-regulating GRP78 and p-AKT/mTOR.

Therefore, our data suggest that down-regulating GRP78 and over-expressing miR-495-3p exerted antitumor effects by reversing chemoresistance through various mechanisms, which provides a possible strategy for treating TNBC.

## Conclusions

In summary, we developed a pirarubicin-resistant TNBC cell line MDA-MB-231R; chemo-resistant cells and tissues over-expressed GRP78, but under-expressed miR-495-3p. In addition, our results revealed that siGRP78 and miR-495-3p mimics inhibited proliferation and reversed chemotherapy resistance; they could therefore become new therapeutic reagents targeting TNBC. The miR-495-3p down-regulation and GRP78 over-expression increased phosphorylated AKT and mTOR levels, while the opposite suppressed p-AKT/mTOR signaling. Based on these results, miR-495-3p might reverse the elevated GRP78 expression in chemo-resistant TNBC by regulating p-AKT/mTOR signaling. Thus, the miR-495-3p mimics suppress the oncogenic function of TNBC by binding to GRP78, and the pirarubicin resistance mechanism involves an accessible signaling pathway (miR-495-3p-GRP78-pAkt/mTOR) as a therapeutic target.

## Supplementary Material

Supplementary Figures S1-S2

## Data Availability

All data generated or analyzed during this study are included in this published article.

## Abbreviations

CCK-8: cell counting kit-8
DFS: disease-free survival
EMT: epithelial–mesenchymal transition
HER-2: human epidermal growth factor receptor 2
MDR: multi-drug resistance
TNBC: triple-negative breast cancer
