# Supplementary material for: Down-regulating GRP78 reverses pirarubicin resistance of triple negative breast cancer by miR-495-3p mimics and involves the p-AKT/mTOR pathway
Source: Biosci Rep. 2022 Jan 6;42(1):BSR20210245. doi: 10.1042/BSR20210245 (PMC8738866; doi:10.1042/BSR20210245)
Supplement: Supplementary Figures S1-S2 [file BSR-2021-0245_supp.pdf]

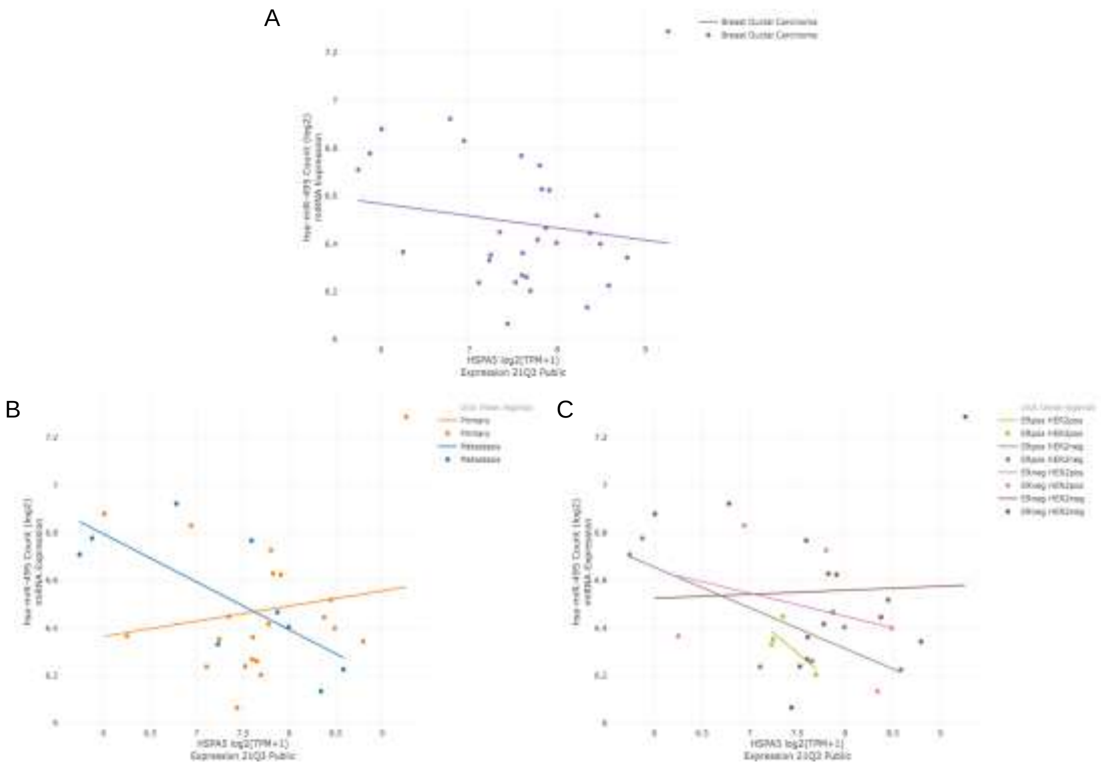
A
Breast Ductal Carcinoma
Breast Ductal Carcinoma
HSPA5 log2(TPM+1)
Expression 21Q2 Public
B
Primary
Primary
Metastasis
Metastasis
HSPA5 log2(TPM+1)
Expression 21Q2 Public
C
HSPA5 log2(TPM+1)
Expression 21Q2 Public

Supplementary Figure 1. GRP78 and miR-495 expression in breast cancer cell lines by searching CCLE database. (A) There was the negative correlation between GRP78 and miR-495 expression in breast ductal cancer cell lines. (B) There was the negative correlation between GRP78 and miR-495 expression in metastatic breast cancer cell lines, not in primary cell lines. (C) The correlation of GRP78 and miR-495 expression in different lineage subtype of breast cancer.

Binding Site: **mirSVR score: -1.1437**
phastcons score: 0.7212

```
hsa-miR-495-3p         3'  uucUUCAC - GUGG - - - - -  UACAAACAAa  5'
                              | | | | |   | | | |       | | | | | | | | |
HSPA5 3´UTR  314:  5'  cauAAGUGACACCAAUAAAUGUUUGUUa  3'
```

Supplementary Figure 2. MiR-495-3p targeting GRP78 3'-UTR via searching miRDB and TargetScan databases
